# The Mediating Role of Job Strain in the Transformational Leadership–Safety Behavior Link: The Buffering Effect of Self-Efficacy on Safety

**DOI:** 10.3390/ijerph16081425

**Published:** 2019-04-21

**Authors:** Byung-Jik Kim, Se-Youn Jung

**Affiliations:** 1 Sogang Business School, Sogang University, Seoul 04107, Korea; kimbj82@business.kaist.edu; 2 College of Business, Korea Advanced Institute of Science and Technology, Seoul 02455, Korea; 3 Prime College, Korea National Open University, Seoul 03087, Korea

**Keywords:** transformational leadership, safety behavior, job strain, self-efficacy regarding safety, moderated mediation model

## Abstract

Although some previous studies have examined the impact of transformational leadership on safety behavior, those works have paid relatively less attention to the intermediating role of employees’ job strain in the link as well as contingent variables that moderate the relationship. Considering that not only job strain substantially affects employees’ perceptions, attitudes, and behaviors in an organization, but also there are some contextual factors that moderate the relationships, we investigated intermediating mechanisms (i.e., mediator and moderator) in the relationship between transformational leadership and safety behavior. Relying on the context-attitude-behavior framework, we conducted a structural equation modeling analysis with a moderated mediation model. Specifically, we hypothesized that the level of an employee’s job strain would mediate the transformational leadership–safety behavior link. We also hypothesized that an employee’s self-efficacy regarding safety would moderate the association between job strain and safety behavior. Using survey data from 997 South Korean employees, we found that all of our hypotheses were supported. The findings suggest that the level of an employee’s job strain mediates and elaborately explains the transformational leadership–safety behavior link. Moreover, an employee’s self-efficacy regarding safety is a buffering factor which decreases the harmful effects of job strain on safety behavior.

## 1. Introduction

Accidents at work have been considered as an important topic due to their profound impact on human life at the physical, mental, and economic levels. Accidents at work function as a fatal blow not only to the victims, but also to their firms and the national economy. The mental and physical damages caused by accidents at work usually make the daily life of the victims devastating for a lifetime. Furthermore, companies with accidents at work are more likely to suffer serious economic losses and long recovery periods. Therefore, efforts to reduce accidents at work are highly required to protect employees’ well-being as well as firms’ sustainability.

According to Bird and his colleague’s domino theory [1], before the occurrence of an accident, a “precursor” appears which functions as the direct cause of the accident. Among various precursors, employees’ unsafe behavior (e.g., impulsive or careless behavior) has been regarded as one of the most direct antecedents of an accident. To enhance the quality of employees’ safety behavior, previous studies have suggested various factors influencing safety behavior. For example, the job demand–resources (JD-R) model [2,3] suggests a useful conceptual model which describes the impact of various physical, psychological, and organizational factors on safety outcomes. The model points out that two types of working conditions including job demands and job resources significantly affect safety outcomes such as safety behavior, accidents, and injuries. The job demands dimension consists of various components that are negatively associated with safety behavior, including a bad physical environment, a high level of work pressure, complexities, and risks. In addition, the job resources dimension consists of various factors that are positively related to safety behavior, including knowledge, autonomy, and a supportive environment (e.g., social support, leadership, a safety climate). 

Among the various antecedents, in this paper, we focus on leadership styles, especially transformational leadership, due to its critical role in explaining various organizational outcomes by affecting employees’ cognitions, emotions, and behavior [4,5,6]. Transformational leadership is defined as a leadership style “broadening and elevating followers’ goals and providing them with confidence to perform beyond the expectations specified in the implicit or explicit exchange agreement” [6]. Although some scholars have investigated the relationship between transformational leadership and employees’ safe behavior [7,8,9], we believe that there are some research gaps to be additionally addressed. 

First, the previous studies which delved into the association between transformational leadership and safety behavior have underexplored the importance of job strain in explaining the intermediating mechanism of the relationship. Job strain can be defined as an employee’s negative perceptions, emotions, and physiological states which emerge when an employee recognizes that he or she cannot adequately deal with various external stimuli such as interpersonal relationships, job characteristics, and the work environment [10,11,12,13]. The concept has been known to critically influence employees’ perceptions, attitudes, and behaviors by diminishing cognitive/emotional/physical abilities to implement his or her tasks and duties. Then, eventually, it deteriorates the quality of his or her performance in an organization [11,12,13]. In other words, job strain of employees functions as a critical construct which not only explains the influence of transformational leadership on employees in an organization, but also predicts their important attitudes or behaviors. Despite its significant theoretical and practical impact on employees in an organization, to the best of our knowledge, there has not been any research to have considered job strain as a critical mediator in explaining the influence of transformational leadership on employees’ safe behavior. Although some previous studies have suggested a bivariate relationship between transformational leadership and job strain [7] as well as between job strain and safety behavior [12], those did not examine the entire relationship among the three variables in an integrated manner based on an overarching theoretical ground. By investigating the intermediating role of job strain with a theoretically overarching basis, we can provide an elaborate explanation on how transformational leadership positively affects safety behavior at work. Thus, examining the role of job strain in describing the association is highly required.

Second, previous studies on the transformational leadership–safety behavior link have paid less attention to the contingent or contextual factors that moderate the relationship. These studies have focused on various intermediators in the link. For example, Shen and his colleagues [9] reported that transformational leadership influences employees’ safety behavior through the sequential mediating roles of safety-specific leader-member exchange (LMX), safety climate, safety knowledge, and safety motivation. Although finding various mediating factors in the relationship between transformational leadership and safety behavior is very important, it is not enough to elaborately describe the influence of transformational leadership on safety behavior because the mediating variables cannot fully explain the association in all situations or contexts. In other words, the explanation of the mediators is limited to a certain situation or context. Thus, we suggest that examining contingent variables (i.e., moderators) in the link would contribute to elaborating the transformational leadership–safety behavior literature. 

To deal with these issues, in the present study, we have investigated the intermediating effect of job strain between transformational leadership and safety behavior, as well as the moderating effect of self-efficacy regarding safety on the job strain–safety behavior link. The theoretical logic is based on the context-attitude-behavior model [14,15], which explains the mediation structure. Grounded on it, we expect that transformational leadership, as one of the important contexts, may build employees’ behavior (i.e., safety behavior) by affecting their attitude (i.e., job strain). Based on previous works, we suggest that transformational leadership would decrease the level of employees’ job strain [7,16,17] and that employees’ job strain would increase safety behavior [12,18,19,20,21]. In the present study, considering that stress indicates changes in well-being because of various stressors while strain means lowered levels of well-being or functioning (e.g., exhaustion, tension, anxiety, and rumination) [11,12,17], we focus on strain of employees at work.

In addition, we propose that there may be contingent factors that moderate the relationship between job strain and safety behavior. Although job strain may diminish the quality of employees’ safety behavior, some buffering factors can weaken the negative influence of job strain on safety behavior. Among those factors, we have focused on employees’ self-efficacy regarding safety since the concept of self-efficacy has been regarded as one of the most fundamental factors which explain an individual’s perceptions, attitudes, and behavior [22,23]. Based on Eden and Zuk’s definition [24], we have defined self-efficacy regarding safety as an individual’s overall estimate or expectation of his or her ability to effectively deal with safety-related situations.

When an employee has a high level of self-efficacy regarding safety, he or she can protect himself/herself from the harmful influences of job strain. Furthermore, the employee may feel that he or she has enough abilities to effectively deal with the harmful effects of job strain at work. Through this, the negative psychological and physical states from job strain would not substantially diminish the quality of safety behavior. On the contrary, if the level of an employee’s self-efficacy regarding safety is low, he or she may feel that he or she cannot adequately deal with many issues from unsafe situations at work. Then, the negative influences of job strain may be facilitated and amplified, being considerably damaging to his or her safe behavior.

To empirically test the above hypotheses, by utilizing data from 997 employees in South Korea, we conducted a structural equation modeling (SEM) analysis with a moderated mediation model.

## 2. Theories and Hypotheses

### 2.1. Transformational Leadership and Job Strain

Some previous works on leadership have shown that transformational leadership decreases the degree of an employee’s job strain [7,16,17]. According to Cummings and Cooper [25], stress can be defined as “the force that causes a strain in the physical and psychological states by escaping from the state of stability”. As the concept of stress develops, there have been a lot of discussions about the concept as well as attempts to describe it in terms of industrial/organizational psychology and organizational behavior theories [11,12]. In the present study, considering that stress indicates changes in well-being because of various stressors while strain means lowered levels of well-being or functioning (e.g., exhaustion, tension, anxiety, and rumination) [11,12,17], we have focused on strain of employees at work. An employee’s job strain has been considered as an important factor which diminishes cognitive/emotional/physical abilities to implement his or her tasks and duties, eventually deteriorating the quality of his or her performance [10,11,12,13]. Based on many previous works on job strain, we have defined job strain as an employee’s negative perceptions, emotions, and physiological states which emerge from recognizing that he or she cannot adequately deal with various external stimuli such as interpersonal relationships, job characteristics, or the work environment [11,12,13,26,27].

Through providing employees with a higher level of inspirational motivation, idealized influence, supportive caring, and intellectual challenges, transformational leadership can reduce various uncomfortable perceptions and emotions in an organization [7,16,17]. Specifically, we suggest that each of the sub-dimensions of transformational leadership may decrease the level of employees’ job strain as follows. 

First, a leader with transformational leadership is likely to provide inspirational motivation to his or her followers. Through this, the followers of the leader may pursue noble values and goals beyond their own ego-centric interests [28,29]. Then, the followers may try to proactively cooperate with their colleagues to achieve novel objectives, which tend to be collective-level. Through this collaborative atmosphere, the followers may feel that they are safe and trusted by colleagues in the organization. Those positive perceptions would decrease their level of job strain.

Second, through idealized influence, a leader with transformational leadership can provide psychological safety to his or her followers, directly contributing to reducing their job strain [28]. The followers of transformational leadership are likely to regard the leaders as their role model, identifying with their leader [30]. Through this identification, the followers would feel that they are competent enough to achieve collective and noble goals like their transformational leader. Their positive perceptions and feelings would alleviate their job strain. 

Third, a transformational leader tends to stimulate employees to think differently with traditional practices. Through this intellectual stimulation, the followers may try to solve various work problems with novel approaches [30]. The new approaches would help the followers to reframe their stressful experiences in positive and effective ways [31]. Furthermore, in the process of stimulation, followers may effectively solve difficult problems in an organization. Their positive experiences would enhance a follower’s sense of self-efficacy, facilitating the more efficient problem-solving ability of followers. By virtue of it, the level of employees’ job strain would be decreased.

Lastly, by providing individual consideration, a transformational leader is likely to reduce employees’ job strain. It is self-explanatory that the leadership style can decrease the level of employees’ strain since the leader’s supportive caring for followers in an individual manner would function as a psychological base for them. The followers may feel that their negative emotions such as anxiety, fatigue, and anger in the process of working are healed and restored, by relying on the psychological base [5,28]. Based on the reasons described above, we suggest that transformational leadership is negatively associated with employees’ job strain in an organization.

**Hypothesis** **1.** *Transformational leadership is negatively related to employees’ job strain.*

### 2.2. Employee’s Job Strain and Safety Behavior

Safety behavior has been considered as one of the most important safe performances in an organization, being defined as employees’ behaviors which seek to prevent mental and physical hazards [12,32,33,34]. Many previous works have reported that safety behavior is closely associated with occupational injuries and accidents in various industries [33,35,36,37]. 

Based on previous studies [12,18,19,20,21], we suggest that job strain would decrease the level of an employee’s safety behavior. As job strain increases, employees may experience functional diminishment in their cognitive/emotional/physical areas [18,19,20]. The loss of such functions has a serious adverse effect on an employee’s abilities pertinent to both attention and prevention for safety [12,18,19,20]. According to the explanation of the stress–thought model, stress not only increases psychological anxiety and physical fatigue, which cause deterioration of cognitive functions, but also makes normal thinking impossible [19,20]. Considering that adequate situational judgments and decision-making processes are essential for conducting proper safety behavior, stress can have a critical and harmful effect on safety behavior. In addition, according to extant works which have explored the relationship between emotions and decision-making processes [38,39], individuals are likely to experience negative emotions when they are under stress. Then, these negative experiences would lower the level of their logical thinking and judgment ability, eventually resulting in impulsive decision-making and unsafe behavior [38,39]. Based on the above studies, we can hypothesize that job strain will reduce the level of employees’ safety behavior.

**Hypothesis** **2.** *An employee’s strain is negatively related to his or her safety behavior.*

### 2.3. Mediating Role of Job Strain between Transformational Leadership and Safety Behavior

As described above, we suggest that an employee’s job strain would mediate the relationship between transformational leadership and safety behavior. Based on the above arguments, we believe that transformational leadership may enhance the level of an employee’s safety behavior by diminishing his or her job strain at work. 

To integrate our hypotheses, which are described above, based on a theoretical ground, we have relied on the context–attitude-behavior framework [14,15] that bolsters the mediation structure. This perspective suggests that a variety of contexts at work (e.g., organizational systems, rules, leadership, and environments) are important preceding factors which significantly affect the attitudes and behavior of employees. Grounded on it, we expect that transformational leadership, as one of the critical contexts, would create employees’ behavior (i.e., safety behavior) by affecting their attitude (i.e., job strain). Previous works theoretically and empirically bolster our hypotheses by demonstrating the negative association between transformational leadership and job strain [7,16,17] and job strain and safety behavior [12,18,19,20,21]. Thus, we hypothesize as follows.

**Hypothesis** **3.** *Employees’ job strain mediates the relationship between transformational leadership and safety behavior.*

### 2.4. Moderating Effect of Employees’ Self-Efficacy regarding Safety between Job Strain and Safety Behavior

We suggest that there may be contingent factors which moderate the relationship between job strain and safety behavior. Although job strain would decrease the quality of an employee’s safety behavior, there may be some buffering factors which can diminish the negative impact of job strain on safety behavior. Among various buffering factors, in this paper, we have focused on employees’ self-efficacy regarding safety because the concept of self-efficacy has been known as one of the most fundamental variables which explain individuals’ perceptions, attitudes, and behavior [22,23]. 

According to Bandura, self-efficacy is defined as the “beliefs in one’s capabilities to organize and execute courses of action required in managing prospective situations. Efficacy beliefs influence how people think, feel, motivate themselves, and act” [22]. Previous works have reported the significant role of self-efficacy in various organizational outcomes [22,23,24]. This concept is considered as a both task-specific and general variable, and it has been known as a dispositional trait that significantly explains individual behaviors across various situations [40]. In this research, we have applied the concept into safety-related contexts. Thus, based on Eden and Zuk’s definition [24], we have defined self-efficacy regarding safety as an individual’s overall estimate or expectation of his or her ability to effectively deal with safety-related situations.

We believe that an employee’s self-efficacy regarding safety can function as a buffering factor that diminishes the negative effects of job strain on the employee’s safety behavior. As described above, job strain would deteriorate the quality of an employee’s safety behavior. The employee’s anxiety and physical fatigue at work which originate in job strain would diminish his or her cognitive functions, directly destroying adequate safe-related decision making. However, if the employee has a high level of self-efficacy regarding safety, he or she can protect himself/herself from the harmful influence of job strain. By virtue of the high-level of self-efficacy, he or she may feel considerable competence in effectively dealing with the harmful effects of job strain. Then, the negative psychological and physical states from job strain may no longer significantly decrease the quality of his or her safety behavior. 

**Figure 1:**
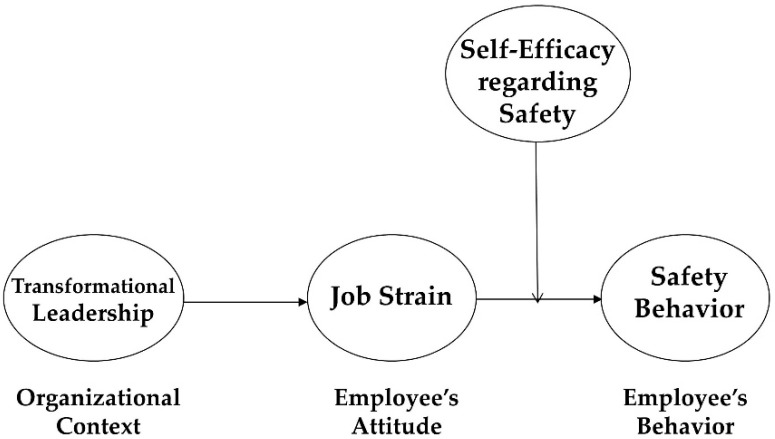
Framework of research model.

In contrast, when an employee has a low level of self-efficacy regarding safety, he or she may feel that he or she cannot deal with various problems from unsafe situations at work (e.g., how to implement safe-related rules and procedures or how to decrease the possibility of safe accidents). In that situation, the negative impact of job strain would be facilitated and amplified, significantly damaging the employee’s cognitive abilities. Then, the quality of safe-related decision-making would be substantially decreased, critically destroying his or her safe behavior. Thus, we propose that an employee’s self-efficacy regarding safety may moderate the relationship between job strain and the employee’s safety behavior (Please see Figure 1).

**Hypothesis** **4:** *An employee’s self-efficacy regarding safety may moderate the relationship between his or her job strain and safety behavior.*

## 3. Method

### 3.1. Data Collection

Considering the residence and industry of the respondents, we chose and contacted companies which had more than 15 employees. Then, considering the size of the company, roughly 3–8 employees were randomly selected. Survey-trained researchers conducted the survey using structured questionnaires. When the quality of the response was bad, the survey was conducted again. Through these processes, data from 997 employees from 103 firms who adequately responded to all the items were utilized in the analysis. The characteristics of the sample are described below (Please see Table 1).

### 3.2. Measures

We measured the research variables with a five-point Likert scale (1 = strongly disagree, 5 = strongly agree). Then, we computed internal consistency of the variables by using Cronbach alpha values.

#### 3.2.1. Transformational Leadership

We utilized 13 items that were adapted from the Multifactor Leadership Questionnaire (MLQ) to measure transformational leadership. The scale was developed by Bass and Avolio [30], consisting of four sub-dimensions: Idealized influence, inspirational motivation, intellectual stimulation, and individual consideration. The 13 items were selected by the suggestions of previous studies on transformational leadership [5,6,7]. Sample items were “the leader in my organization is a role model I want to be” and “my leader articulates a compelling vision of the future”. The Cronbach alpha value was 0.92.

#### 3.2.2. Job Strain

To measure the level of job strain, we utilized 10 items of the job strain scale by adapting the scale of DeJoy and his colleagues [41]. The 10 items of job strain were selected by the authors because those adequately reflected the core components of the measure [41]. Sample items were “I feel nervous and strain because of work” and “I feel nervous when I work”. The Cronbach alpha value was 0.85.

#### 3.2.3. Safety Behavior

We utilized 8 items of the Neal and Griffin’s [36] scale to measure safety behavior. The scale consisted of two sub-dimensions: Safety participation (SP) and safety compliance (SC). Sample items included “I use all necessary safety equipment to do my job” (SC) and “I put in extra effort to improve the safety of workplace” (SP). The value of Cronbach alpha was 0.94.

#### 3.2.4. Self-Efficacy regarding Safety

To measure the level of employees’ self-efficacy regarding safety, we utilized four items by adapting the self-efficacy scale of Bandura [42]. Sample items were “I am confident in reducing the risk of accidents” and “I am capable of maintaining and improving the safety of my workplace”. The value of Cronbach alpha was 0.89.

#### 3.2.5. Control Variables

Considering that various factors can influence employees’ safety behavior [33], we included employees’ gender, tenure, position, and education level in our analysis to control for employees’ safety behavior.

### 3.3. Statistical Analysis

Frequency analysis and correlation analysis were performed using the SPSS 21.0 program. Furthermore, we conducted a structural equation modeling (SEM) analysis by using the Amos 21.0 program. SEM, unlike the existing multiple regression analysis methodology, is capable of “simultaneously” analyzing the direct or indirect path between variables in an integrated model. 

Considering the suggestion of Anderson and Gerbing [43], we took a two-step approach which includes the measurement model and the structural model. To evaluate the model fit of our hypothesized model, various fit indices such as the comparative fit index (CFI), the turker–lewis index (TLI), and the root mean square error of approximation (RMSEA) were utilized. According to previous studies [44,45], when the values of CFI and TLI of a certain model are greater than 0.90 and the value of RMSEA is less than 0.06, then the model can be considered as a good model. Based on this, bootstrapping analysis was conducted to confirm whether the indirect effect of our research model was significant.

## 4. Results

### 4.1. Descriptive Statistics

The descriptive statistics of this research are shown in Table 2. The main research variables including the independent variable, mediator, moderator, and dependent variable were highly correlated.

### 4.2. Measurement Model

To check whether the level of discriminant validity was appropriate, we conducted a confirmatory factor analysis (CFA) for the research variables which were evaluated by the same employee (i.e., transformational leadership, job strain, safety behavior, and self-efficacy regarding safety). The four-factor model had a good fit to the observations (χ2 (df = 140) = 594.71; CFI = 0.965; TLI = 0.958; RMSEA= 0.057). Then, we conducted sequential chi-square (χ2) difference tests to compare the four-factor model with the three-factor, two-factor, and single-factor model, respectively. Specifically, considering that safety behavior and self-efficacy were very highly correlated, we made the two-factor model which loaded the two variables on the same factor. The results of the test showed that the four-factor model had the best fit among all alternative models. Therefore, we believe that the research variables are distinctive (Please see Table 3).

### 4.3. Structural Model

#### 4.3.1. Result of Mediation Analysis

We established a moderated mediation model by utilizing the SEM technique. In the analysis, the association between transformational leadership and safety behavior was mediated by employees’ job strain.

The fit indices of our hypothetical model (Model 1) was good enough: χ2 = 323.22 (df = 77), CFI = 0.960, TLI = 0.945, and RMSEA = 0.057. In the model, all the control variables (i.e., gender, position, tenure, and education level) were not statistically significant. The model demonstrated that transformational leadership was significantly and negatively associated with job strain (β = −0.20, *p* < 0.001), and job strain was significantly and negatively related to safety behavior (β = −0.06, *p* < 0.05). The results suggest that Hypothesis 1 and 2 were supported.

#### 4.3.2. Result of Moderation Analysis

**Figure 2:**
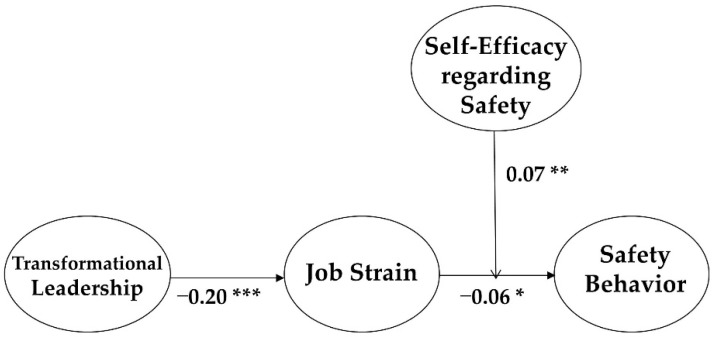
The standardized estimate values of the final model. Notes: * *p* < 0.05, ** *p* < 0.01, *** *p* < 0.001.

To test the moderating effect of employees’ self-efficacy regarding safety, we built a moderated mediation model which simultaneously included both the mediation structure and moderation structure. The moderation effect of employees’ self-efficacy regarding safety on the association between job strain and safety behavior was tested by the model (see Figure 2). As described above, job strain and safety behavior were transformed into mean-centered variables and the interaction term was calculated by multiplying the two transformed variables [46]. Please consider that centered variables are useful in (i) estimating the interaction terms without loss of correlations and (ii) decreasing and testing multicollinearities among research variables. In addition, we tested whether there was a multicollinearity bias between job strain and self-efficacy regarding safety by using the SPSS program. To test this, we calculated the variance inflation factors (VIF) and tolerances [47]. The VIF for job strain and self-efficacy regarding safety was 1.02 and 1.02, respectively, and the tolerance statistics were 0.99 and 0.99, respectively. Because the obtained VIF values were smaller than 10, as well as the tolerance statistics above 0.2, we can conclude that the two variables (job strain and self-efficacy regarding safety) were relatively free from the issue of multicollinearity.

The coefficient of the interaction term (β = 0.07, *p* < 0.01) was significant, implying that employees’ self-efficacy regarding safety functions as a moderator in the association between job strain and safety behavior. In other words, when the level of an employee’s self-efficacy regarding safety is high, the negative effect of job strain on safety behavior is decreased. Thus, the results support Hypothesis 4.

### 4.4. Bootstrapping Analysis

We conducted bootstrapping analysis with a sample of 5000 to test Hypothesis 3, which suggested that there is a mediating effect of job strain between transformational leadership and safety behavior. Note that the mediation effect is significant at a 5% level when the 95% bias-corrected confidence interval (CI) for the mediation effect does not include zero [48]. In the analysis, the bias-corrected CI for the effect on the pathway from transformational leadership to safety behavior via job strain excluded zero (95% CI = (0.01, 0.04)). Thus, the result indicates that the mediation effect of job strain on the path was significant at the level of 5%, supporting Hypothesis 3.

## 5. Discussion

In the present paper, we examined the underlying mechanisms of the relationship between transformational leadership and safety behavior. To empirically test our hypotheses, data from employees in South Korea were utilized. By conducting a moderated mediation model analysis with the SEM technique, we found that employees’ job strain mediated the association between transformational leadership and safety behavior. In addition, employees’ self-efficacy regarding safety functioned as a moderator in the relationship between job strain and safety behavior. In this section, some theoretical and practical implications can be drawn from our results.

### 5.1. Theoretical Implication

We believe that this research may contribute to extending transformational leadership and safety behavior literature by providing these theoretical implications. 

First, we delved into the mediating role of job strain to explain the influence of transformational leadership on employees’ safe behavior. Previous works which examined the association between these variables had paid less attention to the significance of job strain in describing intermediating processes in the link. Considering that employees’ job strain substantially influences employees’ perceptions, attitudes, and behaviors, eventually decreasing the quality of various organizational outcomes [10,11,12,13], our attempt to reveal the important role of job strain as a mediator in the transformational leadership–safety behavior link would be meaningful. Through it, we expect that this research may contribute to elaborating transformational leadership and safety behavior literature.

Second, we found a contingent factor which moderated the relationship between transformational leadership and employees’ safety behavior. Although some previous studies have reported mediators in the link, those have paid less attention to the contingent or contextual factors which moderate the relationship. Of course we acknowledge that investigating mediators in the link would be highly required. However, to elaborately explain the intermediating mechanisms in the transformational leadership–safety behavior link, it is highly required to find certain conditions or contexts under which the mediators work well, since the mediating variables cannot always intermediate the link in all situations. Therefore, we expect that our finding that employees’ self-efficacy regarding safety functions as a buffering factor to decrease harmful effects of job strain on safety behavior would enrich previous works on transformational leadership and safety behavior. 

### 5.2. Practical Implications

We expect that our findings may provide business leaders with practical implications. First, leaders or top management teams who want to enhance employees’ safety behavior through implementing transformational leadership would get some insights from this paper. Our results demonstrated that transformational leadership can increase the quality of an employee’s safety behavior through decreasing his or her job strain. Thus, to check whether their attempt to enhance employees’ safety behavior through facilitating transformational leadership is successful, the top management teams or leaders should monitor the changes in the level of employees’ job strain as an indicator. If the level of job strain has not changed or has even increased, this would indicate that their transformational leadership does not function effectively enough to boost employees’ safety behavior. In addition, the leaders using transformational leadership should focus their leadership behavior on employees’ job strain by implementing “job strain-specific” leadership. 

Second, the findings demonstrated that employees’ self-efficacy regarding safety functions as a buffering factor in the association between employees’ job strain and safety behavior. We expect that the results may emphasize the importance of employees’ individual characteristics in preventing the harmful effects of job strain on safety behavior. As the finding suggests, an employee’s job strain is not always harmful to his or her safety behavior. Its negative impact would be minimized when the employee has a high-level of self-efficacy regarding how to implement safe-related procedures and how to decrease the possibility of safe accidents. Therefore, we suggest that top management teams or leaders should attempt to foster employees’ self-efficacy regarding safety by providing them with safe education programs, safety guidance, and safety systems in an organization.

### 5.3. Limitations and Suggestions for Future Studies

Although we believe that this research has valuable implications from the theoretical and empirical point of view, it has some limitations which need to be addressed. First, in this study, we found that transformational leadership affects safety behavior through “psychological factors” such as job strain. However, prior to such psychological factors, “physical environments” or “physical states of employees” may affect safety behavior. Further studies are needed to verify this. Second, although we conducted a SEM analysis to test our mediation hypothesis, we could not adequately reveal the causal relationships that our research hypotheses claim since this study only utilized cross-sectional data. This should be complemented and alleviated by not only utilizing a longitudinal research design but also by considering the influence of third variables or alternative explanations [49]. In addition, future studies should deal with the fundamental concern that cross-sectional data cannot adequately describe and explain the interaction effects between variables. Third, the data of this study were collected through participants’ self-reports. Since the employee’s behavior which is reflected in the self-report survey may be different from his or her actual behavior, there needs to be adequate supplementation. For example, a third party’s observation or behavioral assessment can be good alternative ways to collect data. Fourth, because the same respondents responded to our survey at the same time, they cannot be free from the common method bias problem. The problem is likely to lead to an overestimation of the correlation between the variables. This limitation needs to be dealt with. Lastly, this paper could not fully utilize the entire items of each measure for our research variables (i.e., transformational leadership and job strain). Although we chose core and essential items from the original version of the measures, future studies should use the full items of the original measures. Fifth, in this study, we only focused on employees’ positive behavior, such as safety behavior, when we investigated the impact of transformational leadership in an organization. However, considering that negative behavior of employees also critically influences organizational outcomes, future studies are needed to deal with negative behavior such as unsafe behavior or counterproductive work behavior. Lastly, this paper did not adequately deal with the issue of nesting of data. Considering that some respondents may be nested into the same organization, the respondents are likely to share the same culture, climate, and leadership style. Thus, the perceptions of the respondents on their transformational leadership tend to be more similar within the organization than between organizations. This research could not deal with this issue. To complement it, additional multi-level approaches are recommended.

## 6. Conclusions

Although this study has various limitations, we believe that it contributes to deepening the transformational leadership and safety behavior literature by investigating a mediating factor between transformational leadership and safety behavior, as well as a contingent factor through which job strain influences safety behavior. Specifically, through this study, we have shown two important findings. First, transformational leadership reduces the level of employees’ job strain, and then the reduced strain ultimately increases their level of safety behavior. Second, although job strain decreases the quality of employees’ safety behavior, their self-efficacy regarding safety functions as a buffering factor by moderating the relationship. The findings show that an employee’s job strain plays an intermediating role in connecting transformational leadership with safety behavior. We also demonstrated the importance of self-efficacy regarding safety in minimizing the negative effects of job strain on safety behavior.

## Figures and Tables

**Table 1:** Descriptive characteristics of our sample.

Characteristic	Percent
**Gender**	
Male	74.8%
Female	25.2%
**Age**	
20s	38.8%
30s	42.5%
40s	15.8%
Above 50s	2.9%
**Education**	
Below high school diploma	35.1%
Community college	16.4%
Bachelor	43.6%
Master’s degree or more	3.4%
**Tenure (in year)**	
Below 5	45.1%
5 to 10	36.2%
10 to 15	10.6%
15 to 20	4.8%
20 to 25	2.6%
Above 25	1.7%
**Firm size**	
Above 1000	8.7%
500–1000 members	14.8%
300–499 members	13.1%
100–299 members	32.6%
50–99 members	25.5%
Below 50 members	5.3%
**Industry Type**	
Manufacturing	53.5%
Transportation	11.0%
Construction	8.9%
Information service and telecommunications	3.2%
Sales	6.9%
Health and welfare	8.6%
Financial/insurance	6.2%
Research and development (R & D)	1.7%

**Table 2:** Means, standard deviations, and correlations between variables.

Variable	Mean	SD	1	2	3	4	5	6	7
1. Gender	1.25	0.44	-						
2. Tenure (year)	6.254	5.66	−0.19 **	-					
3. Position	2.57	0.53	0.29 **	−0.43 **	-				
4. Education level	3.12	0.99	−0.13 **	−0.22 **	−0.14 **	-			
5. Transformational Leadership	3.25	0.64	−0.10 **	0.02	−0.10 **	−0.01	-		
6. Job Strain	2.84	0.66	0.13 **	−0.03	−0.00	0.03	−0.17 **	-	
7. Safety Behavior	3.44	0.78	−0.18 **	0.16 **	−0.10 **	−0.11 **	0.28 **	−0.15 **	-
8. Self-Efficacy regarding Safety	3.37	0.80	−0.24 **	0.19 **	−0.15 **	−0.13 **	0.28 **	−0.12 **	0.74 **

Note: * *p* < 0.01. With regard to gender, male is coded as 1, and female is coded as 2. With regard to position, general manager or higher are coded as 5, deputy general manager and department manager as 4, assistant manager as 3, clerk as 2, and others below clerk as 1. With regard to education, “below high school diploma” level is coded as 4, “community college” level as 3, “bachelor’s” level as 2, and “master’s degree or more” level is coded as 1.

**Table 3:** Chi-square difference tests among alternative measurement models.

Model	χ^2^	*df*	CFI	TLI	RMSEA	Δ*df*	Δχ^2^	Preference
1 Single Factor Model	3653.07	146	0.733	0.687	0.155			
2 Factor Model that integrates (1) TL with job strain, (2) safety behavior with self-efficacy regarding safety	2309.20	145	0.835	0.806	0.122	1	1343.87	2 Factor Model
3 Factor Model that integrates self-efficacy with safety behavior	1092.36	143	0.928	0.914	0.082	2	1216.84	3 Factor Model
4 Factor Model	594.71	140	0.965	0.958	0.057	3	497.65	4 Factor Model

Note: CFI means comparative fit index, TLI means turker–lewis index, and RMSEA means root mean square error of approximation. In addition, Tl means transformational leadership.
